# Predicting acute kidney injury in adults after cardiac surgery with a novel clinical metric: The cardiac diuretic-responsiveness index

**DOI:** 10.1016/j.xjon.2026.101826

**Published:** 2026-04-20

**Authors:** Michael Tyler Guinn, Travis J. Miles, Daniel T. Engelman, Marc R. Moon, Joseph S. Coselli, Todd K. Rosengart, Subhasis Chatterjee, Ravi K. Ghanta

**Affiliations:** a Michael E. DeBakey Department of Surgery, Baylor College of Medicine, Houston, Tex; b Bioengineering Department, Rice University, Houston, Tex; c Department of Surgery, Baystate Medical Center, University of Massachusetts Chan Medical School – Baystate, Springfield, Mass; d Division of Cardiothoracic Surgery, Michael E. DeBakey Department of Surgery, Baylor College of Medicine, Houston, Tex; e Department of Cardiovascular Surgery, The Texas Heart Institute, Houston, Tex

**Keywords:** cardiac surgery, diuretic responsiveness, acute kidney injury

## Abstract

**Objective:**

Cardiac surgery-associated acute kidney injury (CSA-AKI) is a common complication after surgery, yet few risk models are used in practice. We evaluated whether a cardiac diuretic responsiveness index (CDRI) can accurately predict CSA-AKI.

**Methods:**

Time-series hemodynamic, intake/output volumes, medication, and laboratory data were extracted from electronic health records for 2016 patients who underwent cardiac surgery (2017-2022) and linked to the institutional Society of Thoracic Surgeons Baylor St Luke's Medical Center database. Patients with serum creatinine >4 mg/dL, dialysis before surgery, or those without diuretics administered were excluded. CDRI was defined as urine output in the first hour after initial diuretic administration adjusted for patient weight and diuretic dose (cc/kg/hr/mg furosemide). CSA-AKI was defined by Kidney Disease Improving Global Outcomes creatinine criteria. Segmental regression identified the optimal CDRI threshold.

**Results:**

Median time to first dose was 26.4 hours. CSA-AKI occurred in 16.2% (n = 326) of patients who were primarily stage 1 (82.8%, n = 270). Patients who developed CSA-AKI had significantly lower CDRI values (0.0611 vs 0.111 cc/kg/hr/mg furosemide; *P* < .0001). A CDRI threshold of 0.075 predicted CSA-AKI with a C-statistic 0.86), outperforming traditional oliguria criteria (<0.5 mL/kg/h, C-stat 0.8).

**Conclusions:**

CDRI outperforms oliguria in predicting CSA-AKI, enabling real-time stratification, and may inform timely intervention to prevent AKI progression.

**Figure:**
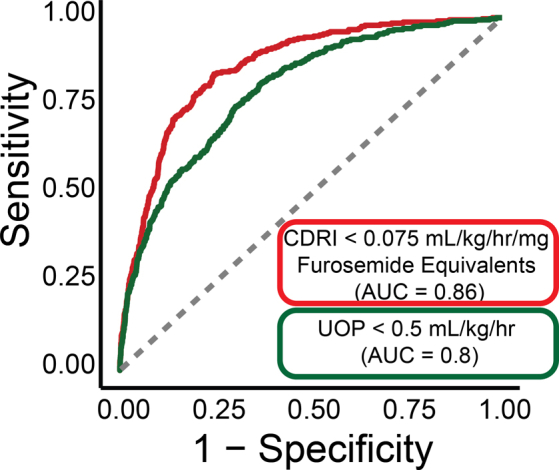
Multivariate model using the cardiac diuretic responsiveness index predicts kidney injury.

**Central Message**

The cardiac diuretic responsiveness index predicts acute kidney injury better than oliguria.

**Perspective**

Diuretics are commonly used after cardiac surgery; however, adequate response is unknown in predicting acute kidney injury. We developed a cardiac diuretic responsiveness index that identifies nonresponding patients and predicts acute kidney injury better than oliguria.

Acute kidney injury (AKI) is common after cardiac surgery, with an estimated incidence ranging from 5% to 30%.^1^, ^2^, ^3^ Moreover, AKI is highly morbid, associated with increased operative mortality, reduced long-term survival, chronic kidney disease development,^4^^,^^5^ and greater costs.^3^^,^^6^, ^7^, ^8^ Currently, AKI is identified through increases in serum creatinine, rather than oliguria. However, this finding is a delayed marker that manifests days after the onset of renal injury. Despite great interest in early AKI detection through urine biomarkers and risk scores, these approaches have not been widely adopted.^9^

Diuretic responsiveness, sometimes referred to as a furosemide stress test, measures urine output after diuretic administration.^10^, ^11^, ^12^ In this test, furosemide (1-1.5 mg/kg) is given to patients with stage I or stage II AKI and urinary output is monitored for 6 to 24 hours for the development of stage II/III AKI. Diagnosis of AKI is the trigger for diuretic administration in patients with an indwelling catheter, laboratory values of renal injury, and who are clinically resuscitated. Although diuretic use and AKI are common after cardiac surgery, data on using diuretic response in AKI prediction remain sparse, mostly in children.^13^^,^^14^ In adults, research has focused on using diuretic responsiveness to predict AKI progression after diagnosis.^15^ Su and colleagues^16^ looked at the furosemide responsiveness index (FRI), the total urine output after the administration of a diuretic in 2 hours normalized by dose, in patients who developed stage I or II AKI within 48 hours of surgery. They found response to furosemide within 24 hours predicted the progression to stage II or III AKI. Another study investigated the modified FRI, which is the FRI normalized to patient weight, which also showed strong discrimination of AKI progression. However, evaluating initial diuretic responsiveness as a predictor of initial cardiac surgery–associated AKI diagnosis after surgery represents a clinically meaningful yet underexplored approach. To address this gap, we created the cardiac diuretic-responsiveness index (CDRI) to predict patients who will develop an AKI, thereby providing clinicians an actionable tool for real-time AKI risk.

## Methods

### Electronic Health Record (EHR) Data Extraction

Data were extracted as previously described.^17^, ^18^, ^19^ In summary, hemodynamic, laboratory, and pharmacologic data were extracted from the EHR platform for patients undergoing cardiac surgery (2017-2022) at Baylor St Luke's Medical Center and were merged with Baylor College of Medicine institutional Society of Thoracic Surgeons data elements. Data were managed in accordance with all institutional data standards and was approved by the Baylor College of Medicine Institutional Review Board with waiver of informed consent (H-44702), approved March 23, 2021.

### Study Population

**Figure E1:**
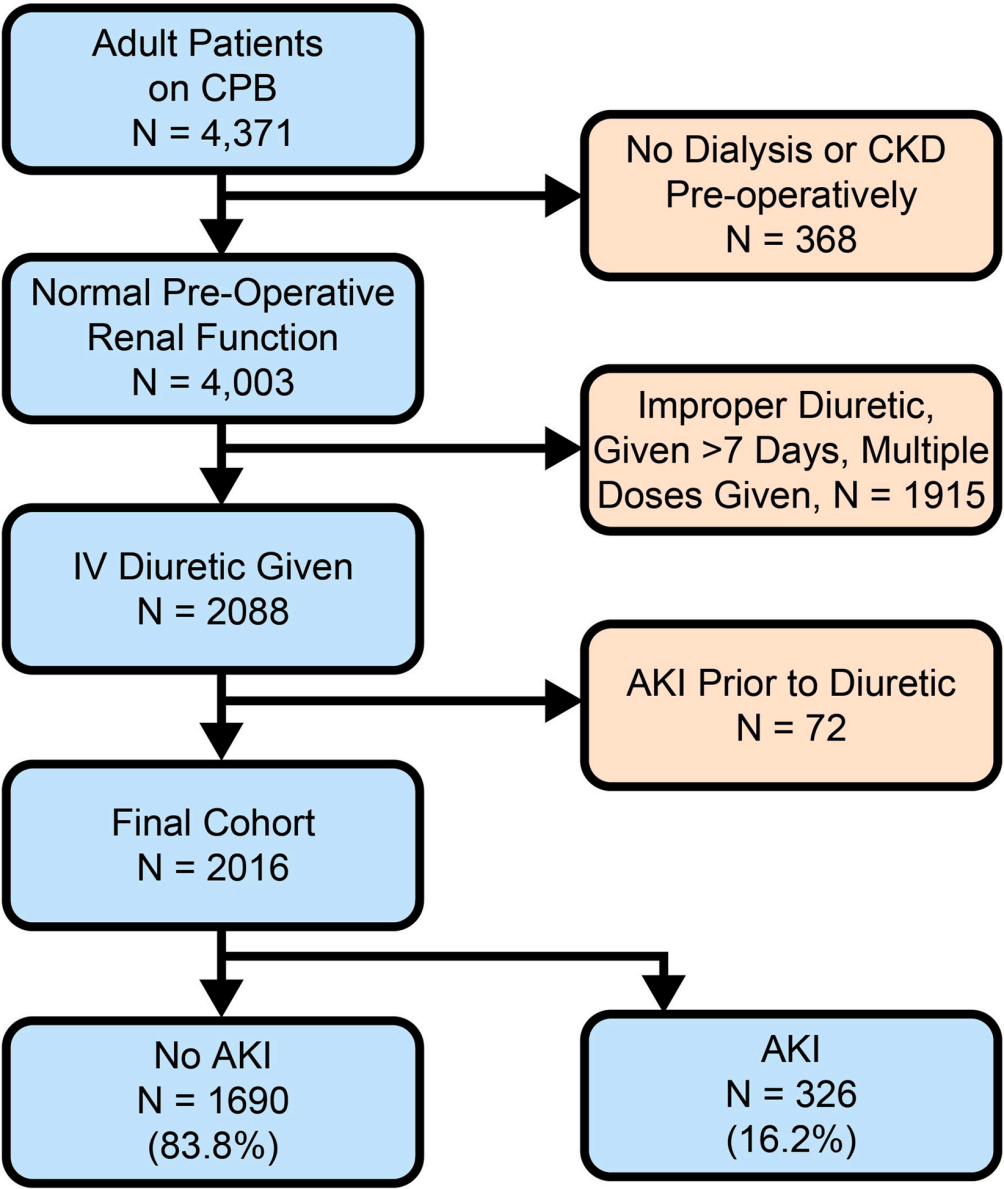
Inclusion criteria for assessing diuretic-responsiveness for urine output production in 6 hours. *CPB*, Cardiopulmonary bypass; *CKD*, chronic kidney disease; *IV*, intravenous; *AKI*, acute kidney injury.

Adult patients (age ≥18 years) who underwent cardiac surgery with cardiopulmonary bypass and received a postoperative intravenous bolus dose of furosemide within 7 days of surgery were identified (Figure E1). Patients with serum creatinine >4 mg/dL, who underwent dialysis before surgery, who developed AKI before the initial diuretic administration, who received continuous diuretic infusions, who received an additional diuretic dose within 1 hour of first dose, or those without diuretics administered were excluded.

### Quantifying AKI and Diuretic Responsiveness

AKI was defined according to the creatinine-based criteria used in the Kidney Disease: Improving Global Outcomes definition.^20^ Baseline creatinine was defined as the creatinine level closest to surgery. Diuretic responsiveness was defined as the urine output in the first hour after the initial furosemide diuretic normalized to patient weight and dose. Additional time intervals were investigated (eg, 2 hours), with similar results. Observed rates of AKI were stratified by diuretic responsiveness using segmental regression demonstrating where the relationship of AKI risk versus CDRI changed most abruptly, defining nonresponders.^21^

### Statistical Analysis

Categorical variables are reported as numbers (percentages) and continuous variables are reported as mean ± standard deviation or median [interquartile range]. Categorical variables were assessed with the χ^2^ test. The Kruskal-Wallis or the Wilcoxon rank-sum test was used to assess significance in univariate analysis. Multivariable logistic regression with the Wald test was used to evaluate the independent effect of CDRI on AKI development adjusting for 25 AKI risk factors identified on univariate analysis. Model strength was estimated through odds ratios (ORs) and 95% CIs (Table E1). All analysis was performed using R Studio software (Posit PBC) with a 2-sided significance level (α 0.05).

## Results

### Study Population Characteristics

A total of 2016 patients without chronic kidney disease who underwent cardiac surgery, ≥18 years, and receiving postoperative furosemide within the first 7 days were included. In this cohort, the patients who did not develop AKI had 911 (53.9%) emergent surgeries, 717 (42.4%) urgent surgeries, and 60 (3.6%) elective surgeries. Median time until first diuretic administration was 26.4 [25.4-27.3] hours. Most patients were started on diuretics on postoperative day 2 (55% [n = 1004]), followed by postoperative day 1 (45% [n = 901]). A total of 326 (16.2%) developed an AKI after the administration of diuretics (Figure E1). Most patients developed stage 1 AKI (82.8% [n = 270]), with 11% (n = 36) and 6.1% (n = 20) developing stage 2 and 3, respectively.

Patients with postdiuretic AKI were older (70 vs 66 years, *P* < .0001), had longer cardiopulmonary bypass times (129 vs 121 minutes), more blood product transfusions (69 vs 46.4%, *P* = .0005), and greater burden of comorbidities (Table E1). Patients who developed a postdiuretic AKI were more likely to have undergone combined coronary artery bypass and valve surgery (10.4% vs 6.4%, *P* = .0175) and less likely to have undergone isolated valve surgery (18.7% vs 29.6%, *P* = .0005). Patients with AKI were more likely to undergo emergent surgery (7.1% vs 3.6%, *P* = .008) and urgent surgery (55.8% vs 42.4%, *P* = .0005) and less likely to undergo elective surgery (36.8% vs 53.9%, *P* = .0005).

Patients with postdiuretic AKI had worse outcomes, including greater rates of in-hospital mortality (8.3 vs 0.6%, *P* < .0001), prolonged ventilation (39 vs 10.9%, *P* = .0005), and stroke (4.3 vs 0.9%, *P* = .0002, Tables 1 and E2). Preoperative aspirin, anticoagulants, and statins were slightly more frequent in patients who developed AKI, whereas beta-blocker use was more frequent in patients without AKI. There was no significant difference in ACE inhibitor or ARB administration between groups (Table E3). We also investigated the hemodynamics, volume status, and vasoactive support at diuretic administration time. We found mean arterial pressure (82 vs 80, *P* > .9), central venous pressure (12.5 vs 11, *P* = .088), and chest tube output as a proxy for bleeding (10 vs 10, *P* = .3) did not differ significantly between groups (Table E4). Lastly, we looked at medication support at the time of diuretic administration. Most patients were not on vasoactive support at the time of diuretic administration. Patients who developed AKI more frequently received norepinephrine (13 vs 4.9%, *P* < .001), epinephrine (8 vs 2%, *P* < .001), and dobutamine (0.9 vs 0.1%, *P* = .033).

**Table 1:** Postoperative outcomes for patients who did and did not develop an AKI

Characteristic	No AKI	AKI	*P* value
Total	1690	326	
Discharge mortality	10 (0.6%)	27 (8.3%)	<.0001
Chronic renal dialysis	7 (0.4%)	50 (15.3%)	<.0001
Postoperative intubation	77 (4.6%)	54 (16.6%)	.0005
CNS stroke	16 (0.9%)	14 (4.3%)	.0002
Prolonged ventilation	185 (10.9%)	127 (39%)	.0005
Sternal infection	17 (1%)	6 (1.8%)	.6855
Reoperation for bleeding	57 (3.4%)	28 (8.6%)	.0185

*AKI*, Acute kidney injury; *CNS*, central nervous system.

### Diuretic Responsiveness and AKI

**Figure 1:**
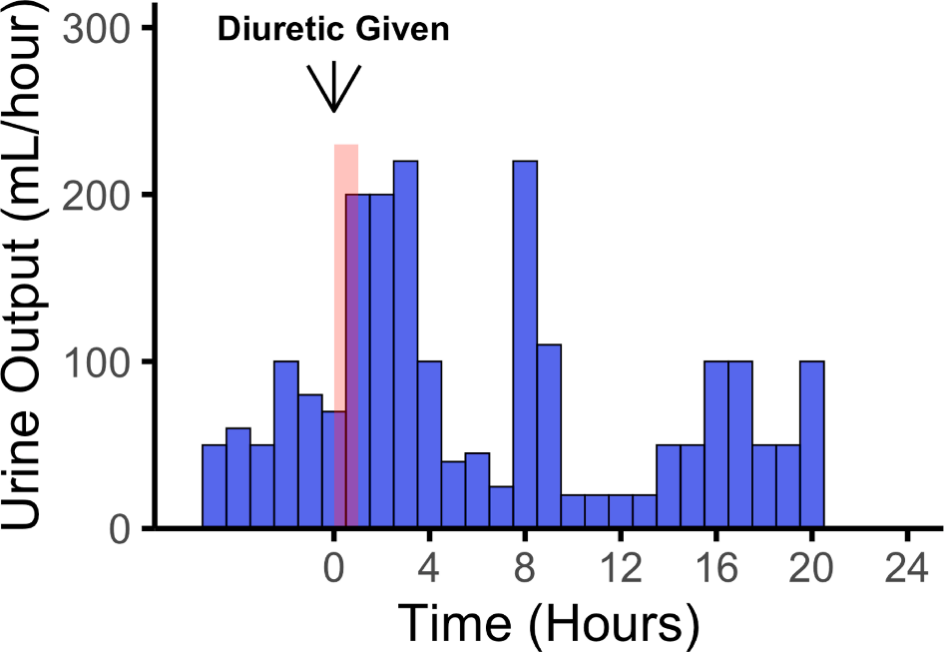
Urine output in response to diuretic. Representative urine output for single patient. The *red area* was used to calculate responsiveness of diuretic (1 hour).

**Figure 2:**
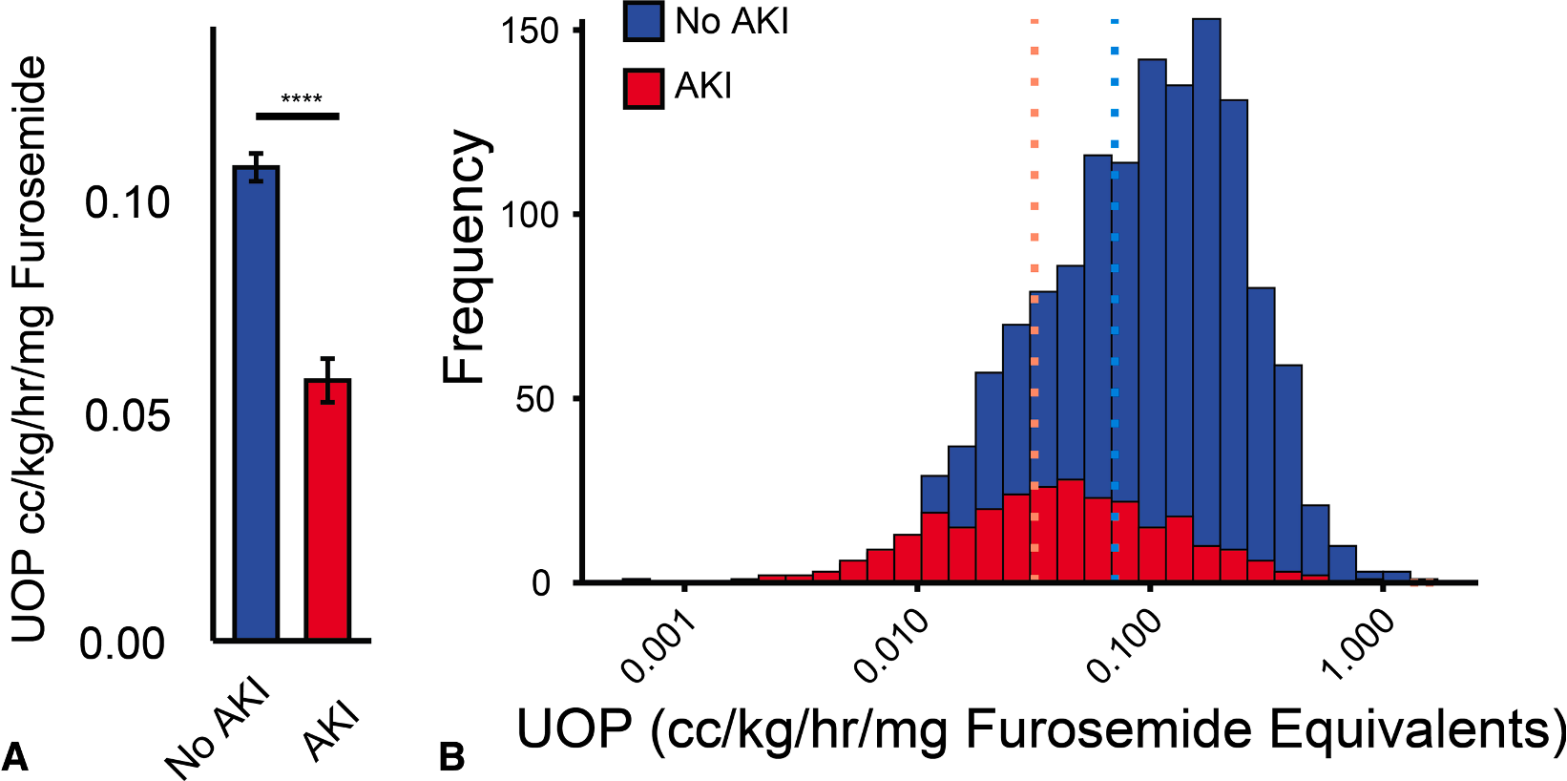
Urine production in 1 hour per weight per milligram of IV diuretic equivalents. A, Mean urine production per hour per kg per mg of IV furosemide equivalents. *Error bars* represent standard error and ∗∗∗∗ represents *P* value < .0001 from Wilcoxon rank-sum test. B, Histogram of population urine production per hour per kg per mg of IV furosemide. *Red/blue histograms* indicate AKI/non-AKI cohorts; *dotted lines* indicate respective group medians. *IV*, Intravenous; *AKI*, acute kidney injury; *UOP*, urine output production.

**Figure E2:**
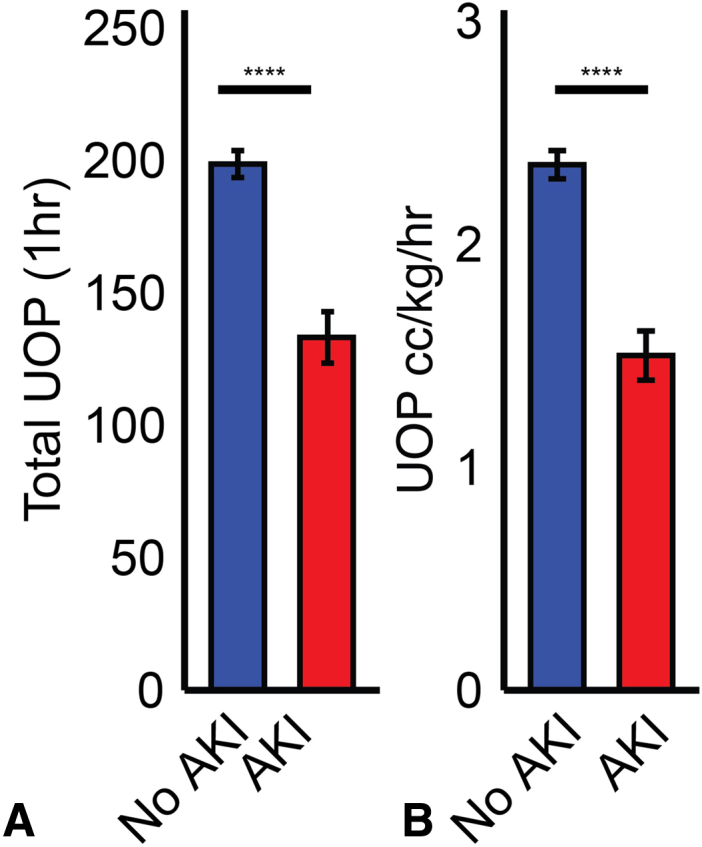
A, Mean total urine production in 1 hour of patients who developed an AKI versus those who did not. *Error bars* represent standard error and ∗∗∗∗ represents *P* < .0001 from Wilcoxon rank-sum test. B, Mean urine production per hour per kilogram of patients who developed an AKI versus those who did not. *Error bars* represent standard error and ∗∗∗∗ represents *P* < .0001 from Wilcoxon rank-sum test. *AKI*, Acute kidney injury; *UOP*, urine output production.

We investigated the urine output production (UOP) normalized to body weight (kilograms) and furosemide dose for every patient (Figure 1). The average UOP in 1 hour and UOP/hour/kg was 188 ±4.58mL and 2.22 ±0.056mL/kg/hr, respectively. The AKI cohort had lower UOP in 1 hour (133 ±9.72 mL vs 199 ±5.09 mL, *P* < .0001) and UOP/hour/kg (1.5±0.11mL/kg/hr vs 2.36±0.063 mL/kg/hr, *P*<.001) (Figure E2). After adjusting for diuretic dose, the average diuretic responsiveness was 0.103±0.0029 cc/kg/hr/mg furosemide. Patients who developed AKI after diuretic administration had significantly lower CDRI (0.0611 ± 0.0051 vs 0.111 ± 0.0033 cc/kg/hr/mg furosemide, *P* < .0001) (Figure 2).

**Figure 3:**
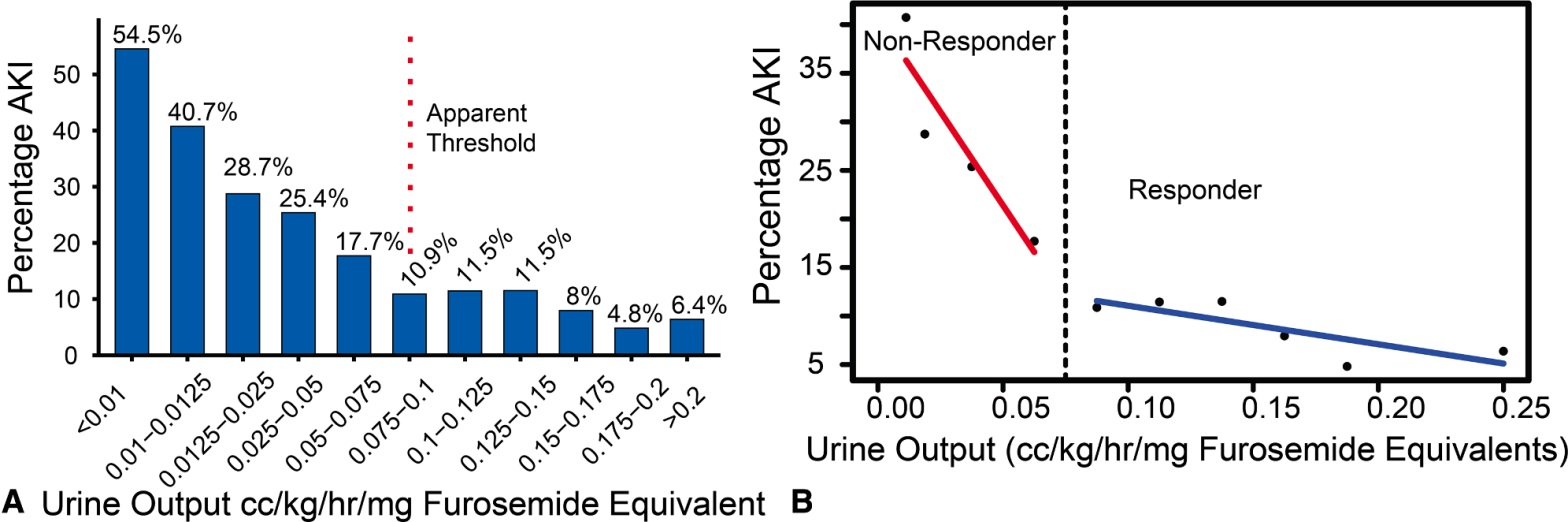
Identifying urine production per hour per kg per mg of IV furosemide equivalent threshold of patients who developed an AKI. A, Patients binned into various urine production per hour per kg per mg of IV furosemide equivalent levels plotted as a percentage of AKI development. The *dotted red line* represents apparent change in slope of AKI development with percentages of AKI per bin over the bar graphs. B, Segmental regression plot showing optimized threshold where slope of AKI development versus diuretic responsiveness found to be 0.075 represented by the *dotted black line*. Patients above the threshold are labeled responders and below are labeled nonresponders. *Black dots* represent percent AKI development in each bin. *IV*, Intravenous; *AKI*, acute kidney injury.

**Figure 4:**
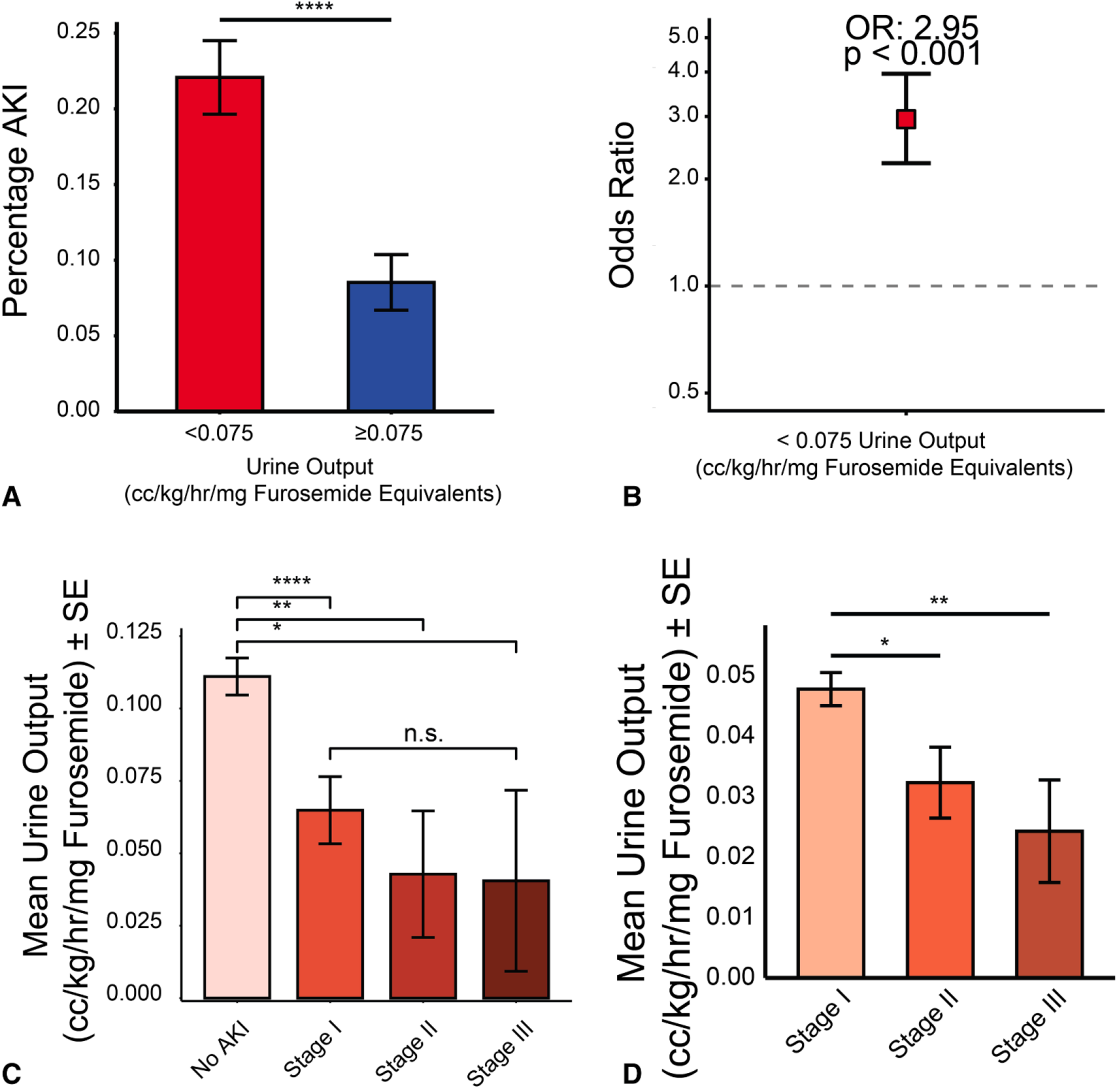
AKI development with clinical threshold. A, Mean percent of AKI development using 0.075 cardiac diuretic responsiveness index (*CDRI*) expressed as urine volume per weight per hour per milligram of IV diuretic equivalents. *Error bars* represent 95% CIs and ∗∗∗∗ represents *P* < .0001 from Wilcoxon rank-sum test. B, OR of AKI development when CDRI is <0.075 compared with those with ≥0.075. Error bars represent 95% CIs. C, Urine production per 1 hour expressed as AKI stage. ∗*P* value < .05, ∗∗*P* < .01, and ∗∗∗∗*P* < .0001. *Error bars* are standard error of the mean. D, Urine production in 6 hours expressed as AKI stage. ∗*P* < .05, ∗∗*P* < .01, and ∗∗∗∗*P* < .0001. *Error bars* are standard error of the mean. *AKI*, Acute kidney injury; *IV*, intravenous; *OR*, odds ratio.

AKI as a function of diuretic responses revealed a gradual increase in AKI incidence with lower diuretic response. Segmental regression analysis revealed a threshold of <0.075 cc/kg/hr/mg furosemide for defining “nonresponders” with a sharp increase in AKI probability (Figure 3). At diuretic responses <0.01 cc/kg/hr/mg, AKI development was 54.5%, whereas responses >0.2 cc/kg/hr/mg were 6.4%. Nonresponders experienced greater rates of AKI (22.1% vs 8.53%, *P* < .0001, Figure 4).

Lastly, to investigate whether diuretic responsiveness could predict AKI evolution, we stratified patients into stage I, II, and III AKI (Figure 4). CDRI at 1 hour distinguished diuretic responsiveness among the AKI stages (Figure 4). Stage I, II, and III had an average CDRI of 0.0478 ±0.00274, 0.0323 ±0.00587, and 0.0243 ±0.00848 cc/kg/hr/mg furosemide, respectively.

### Multivariable Analysis

**Figure E3:**
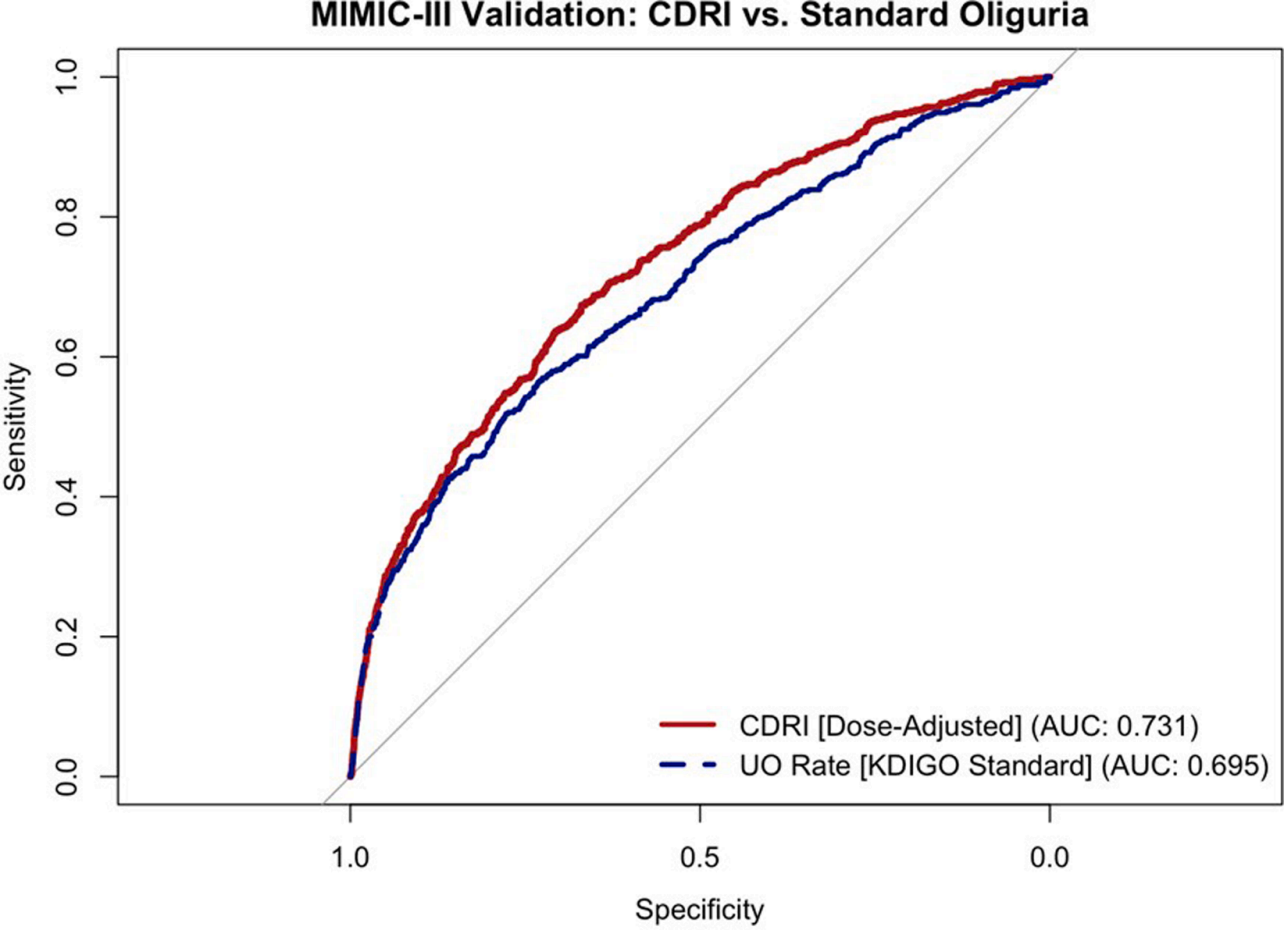
External validation of CDRI versus oliguria in the MIMIC-III database, illustrated by the AUC. *CDRI*, Cardiac diuretic responsiveness index; *MIMIC*, Medical Information Mart for Intensive Care; *AUC*, area under the receiver operating characteristic; *KDIGO*, Kidney Disease Improving Global Outcomes.

**Figure E4:**
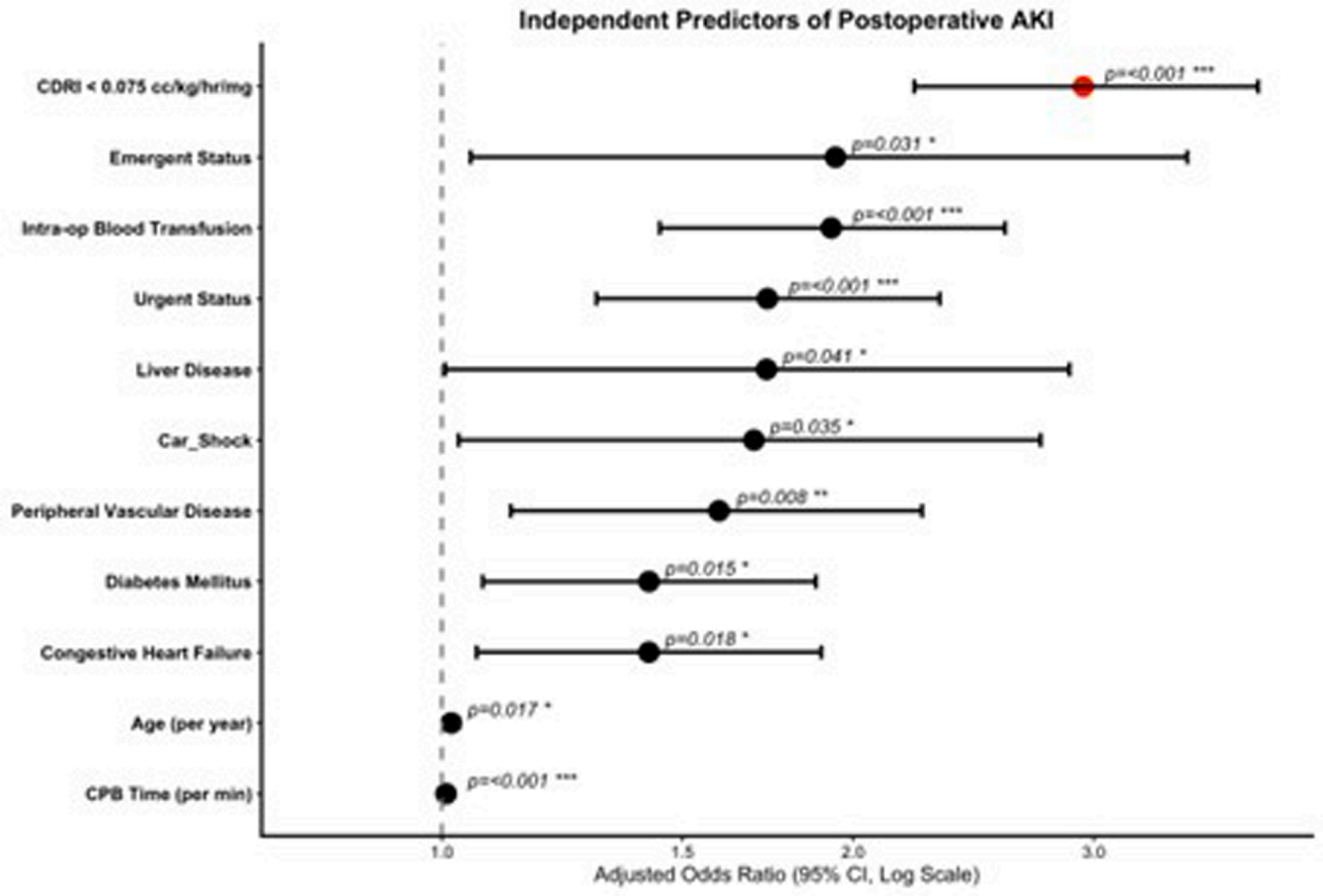
Forest plot of significant variables found on multivariable logistic regression analysis. *Dots* represent odds ratio and error bars are 95% CIs. ∗*P* < .05, ∗∗ is *P* < .01, and ∗∗∗*P* < .001. *AKI*, Acute kidney injury; *CDRI*, cardiac diuretic responsiveness index; *CPB*, cardiopulmonary bypass.

**Figure E5:**
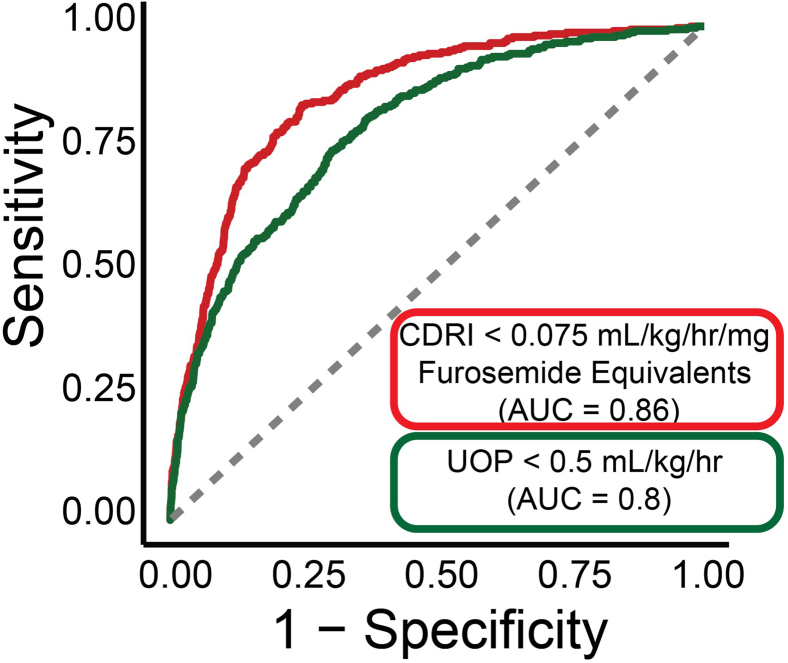
Multivariate analysis of AUC for cardiac diuretic responsiveness index (*CDRI*) versus positive control of < 0.5 mL/kg/h. *AUC*, Area under the receiver operating characteristic; *UOP*, Urine output production.

The variables independently associated with postdiuretic AKI included age (OR, 1.02; *P* = .017), diabetes (OR, 1.42; *P* = .015), congestive heart failure (OR, 1.42; *P* = .018), cardiogenic shock (OR, 1.69; *P* = .035), peripheral vascular disease (OR, 1.59; *P* = .008), liver disease (OR, 1.73; *P* = .041), urgent surgical status (OR, 1.73; *P* < .001), emergent surgical status (OR, 1.94; *P* = .031), cardiopulmonary bypass time (OR, 1.01; *P* < .001), blood products given intraoperatively (OR, 1.93; *P* = .001), and UOP <.075 cc/kg/hr/mg furosemide equivalent (OR, 2.95; *P* < .001, Table E5 and Figure E3). Mean arterial pressure, central venous pressure, and vasoactive support at diuretic administration were not significant in the multivariable model. Multivariate logistic regression model of the CDRI predicted cardiac surgery–associated AKI with high accuracy, outperforming traditional oliguria criteria (C-stat 0.86 vs 0.8, Figure E4). Lastly, to externally validate the CDRI, we looked at the Medical Information Mart for Intensive Care-III database to compare oliguria versus the CDRI, which showed improved AKI prediction like our dataset (Figure E5).^22^

## Comment

This study showed lower diuretic responsiveness predicts AKI development, and a threshold of 0.075 cc/kg/hr/mg can define an adequate response. The CDRI can be implemented at bedside, and patients below the threshold may benefit from perioperative AKI risk-reducing strategies. Recent work has validated that amino acid infusions can reduce AKI in patients who undergo cardiac surgery^23^^,^^24^ by recruiting renal functional reserve and improving oxygenation.^25^^,^^26^ In addition, natriuretic peptide, dexmedetomidine, or fenoldopam can act as renal vasodilators or increasing renal blood flow.^12^ Using the CDRI may identify patients who may benefit from these intervention; however, additional studies will be needed to confirm.

Existing cardiac surgery AKI risk calculators suffer from reliance on unmodifiable preoperative risk factors such as age, limiting utility for postoperative management.^27^, ^28^, ^29^, ^30^ Incorporating intra- and postoperative variables such as crossclamp time and medications can improve accuracy.^31^

In addition, few tools predict renal injury before its manifests on laboratory test. Although urinary biomarkers such as γ-glutamyl transpeptidase and machine-learning methods show promise for AKI prediction, their adoption has been limited as a result of cost, interpretability, and workflow disruptions.^32^, ^33^, ^34^, ^35^, ^36^ In contrast, our study introduces a practical, affordable, and clinically grounded method for early postoperative renal assessment via diuretic responsiveness. Here, administration was ∼1 day after surgery, consistent with reported furosemide response testing being most informative within 24 to 48 hours of dosing. Future studies could investigate preemptive administration for high-risk patients but must be weighed against ongoing clinical features (eg, prolonged bypass time) to determine optimal timing.

To effectively implement the CDRI in the real world, surgeons will need to establish multidisciplinary protocols with critical care teams to calculate CDRI at the bedside and allow system alerts for low values. The CDRI should be considered one element in comprehensive AKI risk management and not a standalone tool. In addition, diuretics used for calculating the CDRI can carry a risk of nephrotoxicity, and the CDRI does not mandate diuretic administration but assesses patients who have already received drugs. With this in mind, we developed an online application (https://cdri-calculator-aki-prediction.streamlit.app/) to allow clinicians to rapidly assess diuretic responsiveness by entering 3 patient parameters. Such values could be calculated by intensive care unit nursing staff, entered in the EHR, and automatically prompt nephrology consultation or interventions. In cases of hypoperfusion, the CDRI may guide fluid administration or inotropes strategies to increase perfusion, possibly reversing AKI. By coupling the CDRI with biomarkers and EHR algorithms prompting clinical recommendations, a more comprehensive picture of each patient can be produced.

Most existing literature on diuretic responsiveness and AKI focuses on general critical care populations, with limited attention on cardiac surgery.^12^ Furthermore, previous studies have primarily explored diuretic responsiveness as a tool for assessing AKI progression or guiding the timing of renal-replacement therapy, rather than for predicting AKI onset.^15^ Studies that have evaluated diuretic responsiveness and AKI after cardiac surgery have been conducted in pediatric populations with small sample sizes, limiting generalizability.^13^^,^^14^ In addition, diuretic practices vary by route (oral, intravenous, or infusions), class (eg, loop diuretics, thiazides), dose, or even performing frequent administrations.^37^ Furthermore, initial timing of first diuretic can vary on the basis of clinicians with the first day postoperatively the most common administration time point.^38^ In contrast, this study introduces a standardized approach to diuretic responsiveness, adjusting for patient weight, diuretic type, and administered dose. This allows for broad applicability and reproducibility across clinical settings. We identify a data-driven threshold for adequate response that can be applied quickly to stratify AKI risk after diuretic administration in patients who undergo adult cardiac surgery. Although diuretic use after cardiac surgery is common, in practice, they are used selectively in patients with cardiac function or volume overload. This allows the CDRI to selectively identify patients who are experiencing clinical abnormalities.

### Study Limitations

This study has several key limitations. First, the retrospective design of the study introduces potential confounding bias that cannot be fully mitigated through multivariable adjustment, given the inherent variability among clinical practice. Second, as a single-center study, the generalizability of these results to other institutions remains uncertain. Although the absolute CDRI threshold in this study cohort should be consistent, it may be subject to variation at other institutions; therefore, external validation is necessary to reduce overfitting and strengthen this tool for AKI risk prediction. Ensuring external validation also offers a rich opportunity for performing a prospective study in multiple hospital settings, which can reduce bias, improve data quality, and improve exposure (eg, diuretic) measurements for patients with highly precise tracking (eg, hourly urine output). As a starting point toward external validation, we showed that the CDRI in the Medical Information Mart for Intensive Care-III dataset outperforms AKI prediction compared with oliguria. Third, although we included the most common diuretic (furosemide), we acknowledge other diuretics may be administered. Incorporating other institutional diuretic strategies will need to be studied to further validate the CDRI. Finally, this study relied on data extracted from the institutional EHR, which is for clinical care rather than research and may be subject to imperfect documentation.

## Conclusions

We present a method for quantifying postcardiac surgery diuretic responsiveness to assess subsequent AKI risk. Patients with inadequate response, defined by the CDRI, were at significantly greater risk for developing AKI. This measure offers early insight into renal functional status before AKI becomes clinically apparent, providing a potential window for timely intervention. Intervention in patients below CDRI thresholds could be beneficial if injuries can be reversed prior to lab abnormalities manifesting. However, these domains require further prospective exploration at multiple centers to validate these findings in guiding postoperative renal management.

### Declaration of Generative AI and AI-Assisted Technologies in the Writing Process

During the preparation of this work the authors used Gemini in order to improve the manuscript clarity. After using this tool/service, the authors reviewed and edited the content as needed and take full responsibility for the content of the publication.

## Conflict of Interest Statement

J.S.C. participates in clinical studies and consults for Terumo Aortic, Medtronic, W. L. Gore & Associates, CytoSorbents, Edwards Lifesciences, and Abbott Laboratories and receives royalties and grant support from Terumo Aortic. M.R.M. is a consultant/advisory board member for Medtronic and Edwards Lifesciences. S.C. has served on advisory boards for Edwards Lifesciences, La Jolla Pharmaceutical Company, Eagle Pharmaceuticals, and Baxter Pharmaceuticals. D.T.E. is on the device safety monitoring board for Edwards Lifesciences Transcatheter Valves; the research trial executive steering committees for Alexion, Genentech, and Bayer; and the medical advisory boards for Medela, Arthrex, AtriCure, J&J, Pharmacosmos, and Chugai pharmaceuticals. All other authors reported no conflicts of interest.

The *Journal* policy requires editors and reviewers to disclose conflicts of interest and to decline handling or reviewing manuscripts for which they may have a conflict of interest. The editors and reviewers of this article have no conflicts of interest.

## Figures and Tables

**Table E1:** Preoperative factors and intraoperative factors assessed for affecting AKI development

Characteristic	No AKI	AKI	*P* value
Total patients	1690	326	
Median age, y [Q1, Q3]	66 [58, 72]	70 [60.25, 74.75]	<.0001
Median BMI [Q1, Q3]	29 [25.7, 33.1]	29.6 [25.8, 34.9]	.1287
Median ejection fraction [Q1, Q3]	58 [50, 60]	57 [45, 60.3]	.0571
Cardiopulmonary bypass time	121 [93, 155]	129 [89, 180]	.0051
Aortic crossclamp time	81 [57, 109]	83 [54, 123]	.1700
Male	1190 (70.4%)	230 (70.6%)	1.0000
Hypertension	1505 (89.1%)	306 (93.9%)	.0226
Diabetes	655 (38.8%)	165 (50.6%)	<.0001
Infectious endocarditis	71 (4.2%)	13 (4%)	.8866
Chronic lung disease	263 (15.6%)	71 (21.8%)	.0075
Heart failure	703 (41.7%)	180 (55.2%)	<.0001
Cerebrovascular accident	170 (48.6%)	47 (57.3%)	.3186
Cardiogenic shock	78 (4.6%)	34 (10.4%)	.0005
Arrhythmia	461 (27.3%)	115 (35.4%)	.0055
Peripheral vascular disease	188 (11.1%)	66 (20.2%)	.0005
Liver disease	75 (4.4%)	23 (7.1%)	.0138
Previous cardiac surgery	658 (38.9%)	145 (44.5%)	.1288
CABG alone	819 (48.5%)	166 (50.9%)	.4383
Valve alone	501 (29.6%)	61 (18.7%)	.0005
CAB and valve surgery	109 (6.4%)	34 (10.4%)	.0125
Aortic surgery	252 (14.9%)	65 (19.9%)	.0200
Elective surgery	911 (53.9%)	120 (36.8%)	.0005
Urgent surgery	717 (42.4%)	182 (55.8%)	.0005
Emergent surgery	60 (3.6%)	23 (7.1%)	.0030
Blood products given intraoperative	785 (46.4%)	225 (69%)	.0005

*AKI*, Acute kidney injury; *BMI*, body mass index; *CABG*, coronary artery bypass grafting; *CAB*, coronary artery bypass.

**Table E2:** Clinical outcomes by AKI stage

Outcome	No AKI	Stage I	Stage II	Stage III	*P* value
Total	1690	270	36	20	
Discharge mortality	10 (0.6%)	17 (6.3%)	4 (11.1%)	6 (30%)	.0005
Chronic renal dialysis	7 (0.4%)	27 (10%)	8 (22.2%)	15 (75%)	.0005
Postoperative intubation	77 (4.6%)	41 (15.2%)	5 (13.9%)	8 (40%)	<.0001
CNS stroke	16 (0.9%)	12 (4.4%)	1 (2.8%)	1 (5%)	.0025
Prolonged ventilation	185 (10.9%)	89 (33%)	23 (63.9%)	15 (75%)	<.0001
Sternal infection	17 (1%)	5 (1.9%)	0 (0%)	1 (5%)	1.0000
Reoperation for bleeding	57 (3.4%)	20 (7.4%)	3 (8.3%)	5 (25%)	.0070

*AKI*, Acute kidney injury; *CNS*, central nervous system.

**Table E3:** Preoperative medications for cohort

Characteristic	Overall N = 2016∗	No AKI n = 1690∗	AKI n = 326∗	*P* value†
Preoperative ACEI/ARB (within 48 h)	770 (38%)	655 (39%)	115 (35%)	.2
Preoperative aspirin (within 5 d)	1028 (51%)	834 (49%)	194 (60%)	<.001
Preoperative beta-blockers	1766 (88%)	1492 (88%)	274 (84%)	.034
Preoperative anticoagulants	571 (28%)	450 (27%)	121 (37%)	<.001
Preoperative statins	1040 (52%)	848 (50%)	192 (59%)	.004

*AKI*, Acute kidney injury;*ACEI/ARB*, angiotensin-converting enzyme inhibitor/angiotensin receptor blocker.

∗ n (%).

† Pearson χ^2^ test.

**Table E4:** Hemodynamics at diuretic administration

Characteristics	Overall N = 2016∗	No AKI n = 1690∗	AKI n = 326∗	*P* value†
Mean arterial pressure, mm Hg	80 [72, 91]	80 [74, 89]	82 [60, 103]	>.9
Central venous pressure, mm Hg	12.0 [8.0, 15.0]	11.0 [7.0, 14.0]	12.5 [10.5, 16.0]	.088
Chest tube output, mL/h	10 [5, 25]	10 [5, 20]	10 [10, 35]	.3
Norepinephrine	127 (6.3%)	83 (4.9%)	44 (13%)	<.001
Epinephrine	60 (3.0%)	34 (2.0%)	26 (8.0%)	<.001
Milrinone	1 (<0.1%)	0 (0%)	1 (0.3%)	.2
Dobutamine	5 (0.2%)	2 (0.1%)	3 (0.9%)	.033

*AKI*, Acute kidney injury.

∗ Median [Q1, Q3]; n (%).

† Wilcoxon rank sum test; Pearson χ2 test; Fisher exact test.

**Table E5:** Preoperative, intraoperative, and postoperative UOP tested on multivariable analysis

Variable	Odds ratio	95% CI	*P* value	Sig
CDRI <0.075 cc/kg/h/mg furosemide	2.95	2.22-3.96	<.001	∗
Age, y	1.02	1.00-1.03	.017	†
Body mass index, kg/m^2^	1.01	0.99-1.03	.229	
Ejection fraction, %	1.00	0.99-1.01	.736	
Male	1.19	0.89-1.60	.233	
Hypertension	1.12	0.67-1.95	.680	
Diabetes mellitus	1.42	1.07-1.88	**.015**	†
Infective endocarditis	0.89	0.43-1.73	.748	
Chronic lung disease	0.85	0.62-1.19	.335	
Congestive heart failure	1.42	1.06-1.90	**.018**	†
Cerebrovascular accident	1.28	0.85-1.98	.247	
Cardiogenic shock	1.69	1.03-2.74	**.035**	†
Arrhythmia	1.19	0.88-1.61	.249	
Peripheral vascular disease	1.59	1.12-2.25	**.008**	‡
Liver disease	1.73	1.01-2.88	**.041**	†
Previous cardiac surgery	0.98	0.74-1.29	.900	
Urgent status	1.73	1.30-2.31	**<.001**	∗
Emergent status	1.94	1.05-3.51	**.031**	†
Procedure: valve only	0.70	0.47-1.05	.087	
Procedure: CABG + valve	1.21	0.73-1.96	.458	
Procedure: aortic surgery	1.11	0.70-1.74	.658	
Cardiopulmonary bypass time, min	1.01	1.00-1.01	**<.001**	∗
Aortic crossclamp time, min	1.00	0.99-1.00	.072	
Intraoperative blood transfusion	1.93	1.44-2.58	**<.001**	∗
Mean arterial pressure, mm Hg	1.01	0.90-1.12	.883	
Central venous pressure, mm Hg	1.09	1.00-1.19	.057	
Chest tube output, mL/h	1.02	0.99-1.05	.199	

Bolded values are statistically significant. *UOP*, Urine output production; *CDRI*, cardiac diuretic responsiveness index; *CABG*, coronary artery bypass grafting.

∗ *P* < .001.

† *P* < .05.

‡ *P* < .01.
